# Radioprotection redefined: drug discovery at the intersection of tardigrade biology and translational pharmacology

**DOI:** 10.3389/fphar.2025.1713914

**Published:** 2025-11-18

**Authors:** Zhitao Cui, Cong Lin, Huiying Zhao, Xiaohui Wang

**Affiliations:** 1 Department of Geriatrics, the First Hospital of Jilin University, Changchun, Jilin, China; 2 Interdisciplinary Laboratory for Frontier Chemistry, Changchun Institute of Applied Chemistry, Chinese Academy of Sciences, Changchun, Jilin, China; 3 School of Applied Chemistry and Engineering, University of Science and Technology of China, Hefei, Anhui, China

**Keywords:** tardigrada, extremotolerance, ionizing radiation, radioprotection, space medicine, drug development

## Abstract

Ionizing radiation inflicts lethal double-strand DNA breaks and oxidative stress that underlie acute radiation syndrome, secondary malignancies, and dose-limiting toxicity in radiotherapy; yet the conventional armamentarium of radioprotectants—aminothiols, broad-spectrum antioxidants, cytokines, and superoxide-dismutase mimetics—yields only modest benefit because of narrow therapeutic windows, systemic toxicity, and inadequate protection of radiosensitive tissues. In striking contrast, tardigrades (*phylum Tardigrada*) routinely endure exposures beyond 5 kGy by deploying a multifaceted defense repertoire that includes genome-shielding proteins such as damage suppressor (Dsup) and Tardigrade DNA-Repair protein 1 (TDR1), families of intrinsically disordered proteins that vitrify cytoplasm and scavenge radicals, antioxidant pigments acquired via horizontal gene transfer, and exceptionally efficient DNA-repair and redox networks. Viewing radioprotection through a translational pharmacology lens reveals a pipeline of emerging modalities—including recombinant or cell-penetrating proteins, mRNA therapeutics, peptidomimetics, and biomimetic nanomaterials—while also spotlighting critical hurdles of scalable bioprocessing, macromolecule stability, immunogenicity, and targeted delivery. By integrating insights from extremophile biology with cutting-edge drug-discovery platforms, tardigrade-inspired interventions promise to safeguard healthy tissue during cancer treatment, reduce casualties in nuclear accidents, and shield astronauts on deep-space missions, thereby redefining the future landscape of radioprotection and transforming an evolutionary curiosity into a potent arsenal of medical countermeasures.

## Graphical Abstract

**Figure:**
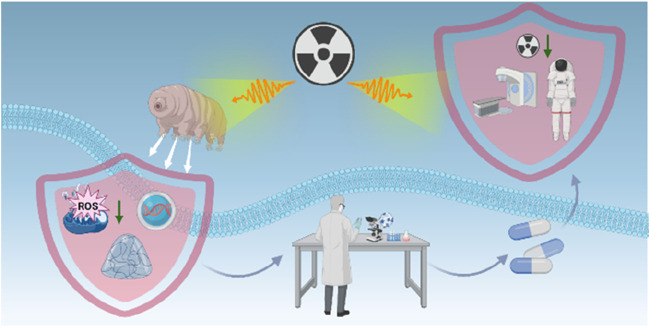
Harnessing tardigrade-inspired radioprotective strategies to revolutionize defense against ionizing radiation in medicine and space exploration.

## 1 Introduction

Ionizing radiation is indispensable to modern society—powering diagnostic imaging, nuclear energy, and, most prominently, cancer radiotherapy (Mahesh, 2013; Gu and Wu, 2023; Sarwar et al., 2025; Taller et al., 2025)—yet the same high-energy photons and particles inflict DNA double-strand breaks, lipid peroxidation, and reactive-oxygen-species (ROS) cascades (Vignard et al., 2013; Santivasi and Xia, 2014; Ye et al., 2020; Bernerd et al., 2022; Jafarian-Dehkordi and Hoeschen, 2025) that culminate in acute radiation syndrome, long-term carcinogenesis, and dose-limiting toxicities (Hou et al., 2024; Talapko et al., 2024; Tao et al., 2024). Damage arises through direct ionization of biomolecules and, more commonly, through hydroxyl radicals generated by water radiolysis, which attack deoxyriboses and bases to create single- and double-strand breaks, abasic sites, and clustered lesions (Jafarian-Dehkordi and Hoeschen, 2025; Rowe et al., 2025); mitochondrial membrane peroxidation further amplifies ROS, triggering checkpoints, apoptosis, senescence, and immune dysregulation (Kawamura et al., 2018; Guéguen et al., 2019; Zhou et al., 2022). An effective radioprotectant must therefore (i) physically shield DNA, (ii) scavenge ROS, (iii) accelerate repair, or (iv) modulate cell-death pathways.

Over 7 decades of research have yielded only a narrow arsenal of agents that meet these criteria marginally. Aminothiols such as amifostine (WR-2721) donate hydrogen atoms to DNA radicals but induce hypotension and nausea, confining their use to niche indications (Weiss and Landauer, 2009). Cytokines (e.g., interleukin-11, granulocyte- and macrophage-colony-stimulating factors) accelerate hematopoietic recovery yet do not prevent the initial molecular insults (Hérodin and Drouet, 2005; Wang et al., 2005). Small-molecule antioxidants and superoxide-dismutase mimetics offer modest free-radical scavenging at tolerable doses, yet their poor nuclear penetration and brief half-lives limit efficacy (Zhang et al., 2016; Yao et al., 2018; Mapuskar et al., 2019; Ivanova et al., 2021; Lim et al., 2023; Liu et al., 2023; Jiang et al., 2025). Collectively, these shortcomings highlight an urgent need for safer, more potent radioprotectants suitable for oncology, spaceflight, and radiological emergencies.

**FIGURE 1:**
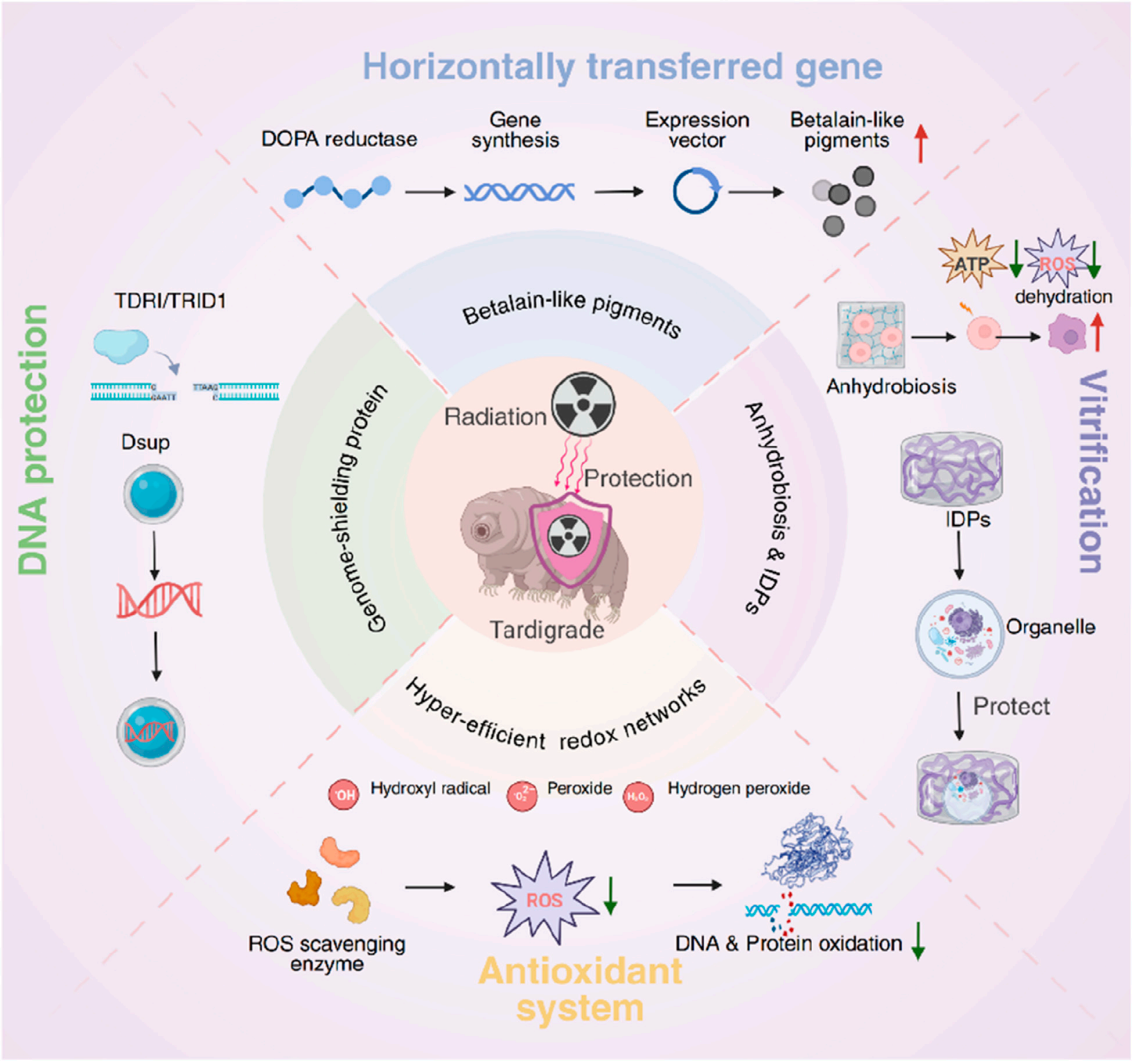
Molecular mechanisms underlying radiation resistance in tardigrades. Key adaptations include: (1) anhydrobiosis, a desiccation-induced ametabolic state; (2) intrinsically disordered proteins that vitrify cellular components and limit radical diffusion; (3) genome-shielding proteins that coat chromatin and reduce DNA damage; (4) distinctive antioxidant pigments that neutralize reactive oxygen species; and (5) hyper-efficient DNA repair and redox systems that rapidly restore genomic integrity post-irradiation.

Tardigrades (*phylum Tardigrada*)—microscopic eight-legged metazoans colloquially termed “water bears”—offer an unparalleled biological blueprint for such innovation (Goldstein, 2022). Numerous species routinely endure γ-ray doses exceeding 5,000 Gy and heavy-ion bombardment beyond 6,000 Gy, orders of magnitude above the human lethal threshold (∼4–5 Gy) (Jönsson et al., 2005; Horikawa et al., 2006; Beltrán-Pardo et al., 2015; Jönsson et al., 2016; Anoud et al., 2024; Clark-Hachtel et al., 2024). Survival is facilitated by a constellation of molecular adaptations: anhydrobiosis, in which cells vitrify and metabolic activity plummets (Rebecchi et al., 2007); intrinsically disordered cytoplasmic, mitochondrial, and secreted abundant heat-soluble proteins that sequester biomolecules and quench free radicals (Chakrabortee et al., 2012; Boothby et al., 2017; Yoshida et al., 2017; Hesgrove and Boothby, 2020; Arakawa and Numata, 2021; Nguyen et al., 2022; Lim et al., 2024); genome-shielding proteins such as damage suppressor (Dsup) (Hashimoto et al., 2016; Hashimoto and Kunieda, 2017) and Tardigrade DNA-repair protein 1 (TDR1) (Chavez et al., 2019; Anoud et al., 2024) that physically coat chromatin; distinctive antioxidant pigments derived from horizontal gene transfer (Li L. et al., 2024); and hyper-efficient DNA repair and redox networks (Jönsson et al., 2005; Kamilari et al., 2019; Clark-Hachtel et al., 2024; Rolland et al., 2024) (Figure 1). Notably, many of these mechanisms evolved to counter desiccation—an environmental stress that, like radiation, induces ROS and strand breaks—highlighting functional convergence that can be co-opted for radioprotection.

Rapid advances in tardigrade genomics, structural biology, and synthetic biology have begun translating these extremophile adaptations into drug-development concepts. Recombinant or cell-penetrating versions of Dsup, mRNA therapeutics encoding stress-tolerant proteins, peptidomimetic derivatives of intrinsically disordered motifs, and biomimetic nanomaterials that emulate vitrification are under active investigation (Hashimoto et al., 2016; Piszkiewicz et al., 2019; Malki et al., 2022; Kirtane et al., 2025). Yet significant hurdles remain, including scalable tardigrade cultivation, stability and immunogenicity of macromolecular protectants, and targeted delivery to vulnerable tissues. Bridging these gaps demands an integrative, translational pharmacology framework that couples extremophile biology with medicinal chemistry, bioengineering, and rigorous preclinical validation. Positioned at the intersection of radiobiology and drug discovery, this perspective integrates the latest insights into tardigrade radiotolerance, critically evaluates the shortcomings of existing radioprotective agents, and delineates a translational roadmap for transforming the unparalleled resilience of these “water bears” into next-generation therapeutics for both Earth-based and space-borne radiation hazards.

## 2 Tardigrade biology and radiation resistance

Tardigrades have emerged as textbook examples of extremotolerance (Bates, 2022), with several species surviving doses of ionizing and ultraviolet radiation that exceed the human lethal threshold by three orders of magnitude (Jönsson et al., 2005; Horikawa et al., 2006; Beltrán-Pardo et al., 2015; Jönsson et al., 2016; Rolland et al., 2024). Their radiotolerance is not an isolated trait but the by-product of a broader survival toolkit honed to withstand desiccation, temperature extremes, osmotic shock, and even the vacuum of space (Rebecchi et al., 2007; Rebecchi et al., 2009; Ito et al., 2016; Tsujimoto et al., 2016; Weronika and Łukasz, 2017; Jönsson, 2019; Saigo et al., 2025). Central to this versatility is the ability to enter anhydrobiosis, a reversible ametabolic state in which the animal contracts into a desiccated “tun,” loses >95% of its body water, and vitrifies its cytoplasm (Rebecchi et al., 2007). During anhydrobiosis, metabolism almost halts (Pigon and Weglarska, 1955), endogenous ROS production plummets (Goldstein, 2022; Sadowska-Bartosz and Bartosz, 2024), and a glass-like intracellular matrix physically immobilizes macromolecules—passively limiting radical diffusion and mechanical damage when radiation strikes (Hengherr et al., 2009; Traspas and Burchell, 2021).

That matrix is built largely from families of intrinsically disordered proteins (IDPs) unique to tardigrades: cytosolic abundant heat soluble protein (CAHS), mitochondrial abundant heat soluble protein (MAHS), secreted abundant heat soluble protein (SAHS), and late-embryogenesis abundant (LEA) proteins. Upon drying, these hydrophilic IDPs self-assemble into an amorphous bioglass that encases DNA, membranes, and enzymes, maintaining native-like hydration shells and preventing denaturation (Chakrabortee et al., 2012; Boothby et al., 2017). Although classical desiccation-tolerant organisms accumulate high trehalose (Erkut et al., 2011; Tapia and Koshland, 2014; Boothby et al., 2017), tardigrades deploy only trace amounts (Hengherr et al., 2008); metabolomic work shows that even low trehalose synergizes with IDPs to strengthen vitrification, indicating a multifaceted, predominantly protein-based strategy (Nguyen et al., 2022).

Beyond passive shielding, tardigrades wield active molecular defenses that remain operative in both hydrated and tun states. The discovery of Dsup, an intrinsically disordered, nucleosome-binding protein, was pivotal: Dsup coats chromatin and cuts X-ray–induced double-strand breaks by ∼40%–50% in human cells engineered to express it (Hashimoto et al., 2016; Hashimoto and Kunieda, 2017; Mínguez-Toral et al., 2020; Zarubin et al., 2023). Orthologues such as TDR1 and recently characterized tardigrade specific radiation induced disordered protein (TRID1) extend this concept, forming phase-separated repair hubs that concentrate DNA repair enzymes at damage sites. Complementing chromatin shields are potent antioxidant systems (Anoud et al., 2024; Li L. et al., 2024; Swamy and Boothby, 2024). Some species synthesize betalain-like pigments via a horizontally transferred L-3,4-dihydroxyphenylalanine (DOPA)-dioxygenase gene (Li L. et al., 2024), while others deploy conventional ROS-scavenging enzymes (superoxide dismutase, catalase, peroxiredoxin) or even secreted fluorescent compounds that absorb ultraviolet (UV) C and re-emit harmless blue light (Moller, 2001; Schokraie et al., 2010; Chavez et al., 2019; Suma et al., 2020; Li L. et al., 2024; Sadowska-Bartosz and Bartosz, 2024). Collectively, these antioxidants quench hydroxyl radicals generated by water radiolysis, reducing indirect DNA and protein oxidation.

Enhanced DNA repair provides a final line of defence. Genomic and transcriptomic surveys reveal expanded repertoires of homologous-recombination and non-homologous end-joining genes, many expressed at constitutively high levels or massively upregulated within hours of irradiation. Tardigrades also appear to bolster mitochondrial NAD^+^ regeneration—via upregulated the mitochondrial chaperone ubiquinol-cytochrome c reductase (bc1) synthesis (BCS1).

BCS1 and NADH dehydrogenase (ubiquinone) 1 beta subcomplex subunit 8 (NDUFB8) assembly factors—to sustain poly (ADP-ribose) polymerase (PARP)-dependent single-strand break repair without exhausting cellular energy reserves. Such metabolic reallocation ensures that even after thousands of gray residual lesions are swiftly and accurately mended, allowing normal reproduction once stress subsides (Clark-Hachtel et al., 2024; Li L. et al., 2024).

These layers—vitrification, chromatin shielding, radical scavenging, and expedited repair—operate in concert, explaining why both hydrated adults and desiccated tuns can survive gamma, heavy-ion, and UV doses lethal to other metazoans. Importantly, field and space-flight experiments have validated this resilience: tardigrades exposed to open space on the FOTON-M3 satellite revived and reproduced after enduring vacuum, cosmic rays, and unfiltered solar UV, and tun-state individuals have withstood shock pressures approaching 1 GPa in impact-survival tests (Chavez et al., 2019). Such demonstrations underscore the translational promise of tardigrade-inspired radioprotectants—whether recombinant Dsup variants, mRNA therapeutics, peptidomimetic IDP motifs, or antioxidant nanomaterials (Malki et al., 2022; Kirtane et al., 2025)—to safeguard human tissues during radiotherapy, nuclear incidents, and deep-space missions. Integrating these extremophile adaptations into drug-discovery pipelines offers a blueprint for fundamentally redefining radioprotection.

## 3 Tardigrade-inspired radioprotective drug development

Tardigrades have emerged as a unique biological model for developing radioprotective pharmaceuticals due to their extraordinary resilience to extreme environments, particularly ionizing radiation (Jönsson, 2019). Their survival capabilities stem from a suite of molecular adaptations—ranging from DNA-protective proteins to metabolic regulators—that are now being translated into therapeutic strategies for human application (Kasianchuk et al., 2023). Central to this endeavor is the identification and engineering of tardigrade-derived biomolecules that can confer radioprotection without compromising safety or efficacy.

One of the most compelling discoveries is the Dsup, a chromatin-associating protein from *Ramazzottius varieornatus* that reduces DNA double-strand breaks upon radiation exposure (Hashimoto and Kunieda, 2017; Mínguez-Toral et al., 2020; Zarubin et al., 2023). Initial efforts to translate this mechanism into human therapy have focused on gene and mRNA delivery systems. In a breakthrough study, Dsup mRNA was encapsulated in polymer-lipid nanoparticles and delivered to mouse mucosal tissues, achieving a ∼50% reduction in radiation-induced DNA damage without affecting tumor tissues—an ideal profile for adjunctive radiotherapy. The transient expression enabled by mRNA circumvents genomic integration risks, while tissue-targeted delivery limits systemic exposure and immune activation (Kirtane et al., 2025). Further work is ongoing to deimmunize Dsup through sequence humanization and peptide engineering, potentially allowing repeated clinical use (Hashimoto et al., 2016). Complementary strategies involve designing cell-penetrating Dsup protein constructs and peptidomimetics that mimic its DNA-binding motifs, offering alternative delivery routes.

Beyond Dsup, additional tardigrade proteins—such as TRID1, which may facilitate DNA repair through phase-separation dynamics, and pigment-biosynthetic enzymes with inherent antioxidant capacity—offer intriguing avenues for exploration (Li L. et al., 2024). Yet interspecies incompatibilities and unpredictable phase behavior in human cells demand thorough functional validation. A complementary strategy is to design humanized constructs that emulate tardigrade mechanisms, for example, by fusing Dsup-like DNA-binding motifs to human chromatin proteins, thereby preserving protective efficacy while minimizing immunogenic risk (Kirtane et al., 2025).

Tardigrade biology also inspires small-molecule radioprotective strategies. Compounds that emulate their antioxidant and DNA repair-enhancing mechanisms, such as NAD^+^ precursors (e.g., nicotinamide, nicotinamide riboside, nicotinamide mononucleotide) and Nrf2 activators, align with the metabolic shifts tardigrades invoke during stress (Li et al., 2017; Zhao et al., 2022; Obrador et al., 2023). Pigments like betalains, known for their antioxidant capacity and radiation absorption, offer dietary supplement potential with low toxicity (Polturak et al., 2017; Huang et al., 2024). Novel small molecules that localize to the nucleus and scavenge free radicals—such as DNA-targeted nitroxides—could also serve as pharmacological analogs to Dsup, though their design must balance protective efficacy with toxicity and mutagenic potential (Soule et al., 2007).

**FIGURE 2:**
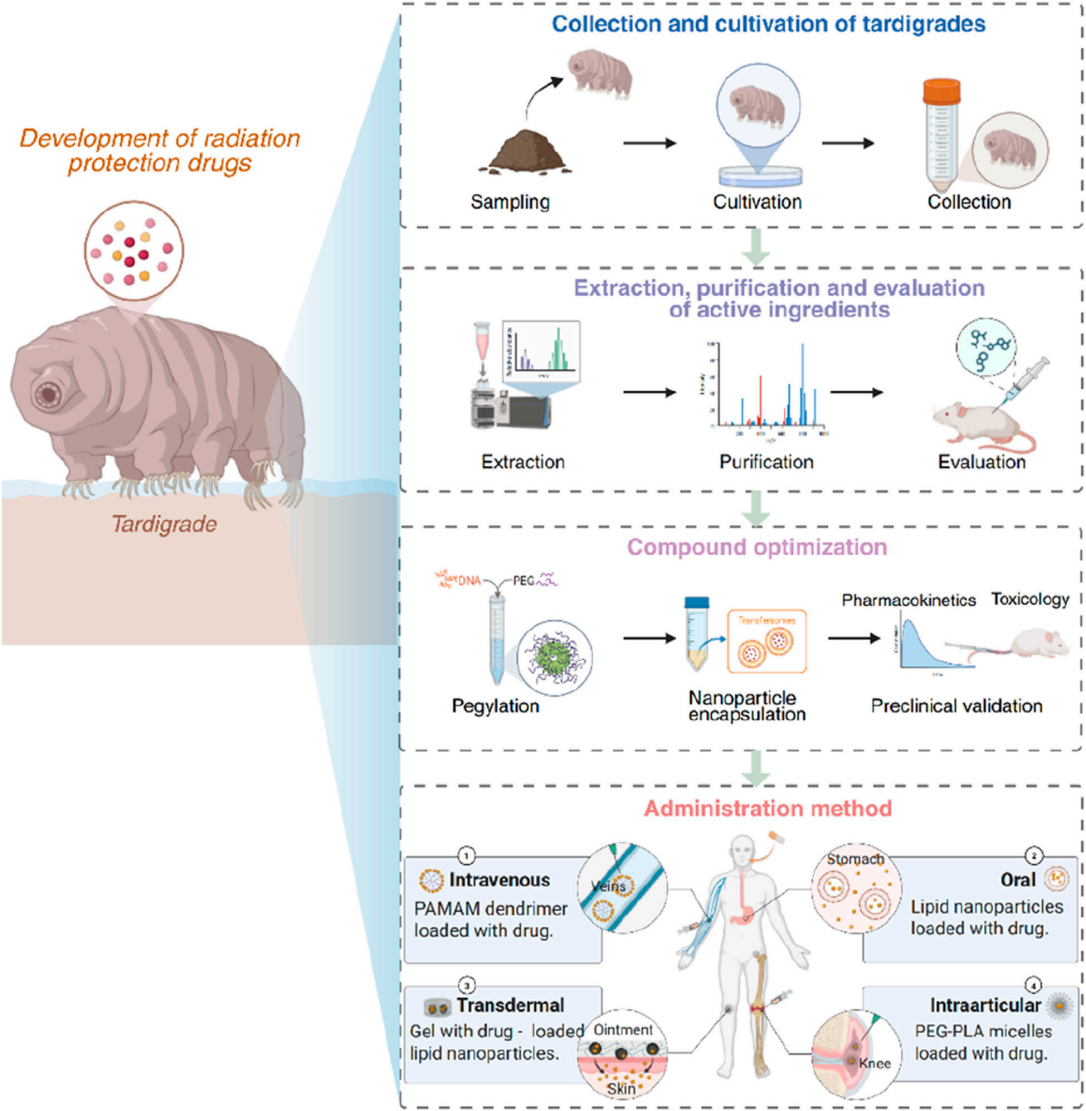
Tardigrade-inspired radioprotective drug development pathway, illustrating key stages: sourcing and culturing of tardigrades, extraction and purification of bioactive components, activity evaluation, compound optimization, and formulation of effective delivery platforms.

To translate these insights into viable drugs, a structured development path is essential. This begins with sourcing and culturing tardigrades at scale (Figure 2), often requiring customized bioreactor systems and stress conditioning to enhance expression of protective molecules (Tsujimoto et al., 2016; Chartrain et al., 2025). Subsequent extraction, fractionation, and bioassay-guided purification (Figure 2)—using models like *Caenorhabditis elegans* and human cell lines—enable the identification of active components. Once lead compounds are identified, their *in vivo* efficacy is evaluated in rodent models of acute radiation syndrome and tissue-specific damage. These tests assess survival, histological preservation, hematopoietic recovery, and DNA damage biomarkers.

Mechanistic studies accompany these evaluations, employing DNA damage assays, oxidative stress measurements, and apoptosis markers to delineate how a candidate functions. For example, Dsup’s DNA protection can be visualized via reduced γ-H2AX foci (Kirtane et al., 2025), while metabolic slowdown induced by CAHS proteins may be monitored through ATP depletion and biostability indicators (Sanchez-Martinez et al., 2024). These mechanistic insights not only guide optimization but inform safety assessments—ensuring that protective agents do not inadvertently shield tumors or disrupt normal DNA damage responses.

Lead optimization involves structure-activity relationship studies to refine small molecules and protein engineering approaches to enhance molecular stability, delivery efficiency, and immunocompatibility. To achieve these goals, strategies such as PEGylation, minimal motif identification (Roces et al., 2020; Akar et al., 2024; Li G. et al., 2024), and nanoparticle encapsulation are commonly employed. Once optimized, candidates undergo comprehensive preclinical evaluation, including assessments of pharmacokinetics, toxicity, dosing regimens, and therapeutic efficacy (Figure 2)—particularly in large animal models when warranted. Delivery platforms are tailored to specific use cases (Figure 2): localized administration methods such as peritumoral injections, oral rinses, and suppositories are preferred for minimizing systemic exposure in contexts like cancer radiotherapy. In contrast, for applications requiring systemic protection—such as during spaceflight or nuclear emergencies—oral formulations, injectable agents, and Dsup-derived therapeutics are under active development (Wu and Schultz, 2020; Kirtane et al., 2025). These innovations are particularly suited to the needs of astronauts exposed to chronic galactic cosmic radiation or civilians affected by radiation-related disasters.

In sum, tardigrade-inspired drug discovery represents a frontier in radioprotective medicine, bridging extremophile biology with human therapeutic innovation. By systematically integrating protein engineering, metabolic modulation, and advanced delivery systems, the field is poised to deliver next-generation radioprotectants for use in oncology, spaceflight, and civil defense.

## 4 Challenges, future directions and potential applications

Developing tardigrade-based therapeutics represents a promising yet complex frontier in biomedical science. Despite groundbreaking discoveries like the Dsup protein and CAHS systems (Piszkiewicz et al., 2019; Kirtane et al., 2025), translating these extremophile-derived mechanisms into safe and effective human interventions involves overcoming substantial technical, regulatory, and conceptual hurdles. One fundamental challenge lies in the cultivation and biomass scale-up of tardigrades themselves. Unlike microorganisms, tardigrades reproduce slowly and require specific culture conditions (Tsujimoto et al., 2016). Innovations such as bioreactors with microcarriers, semi-automated feeding systems, or stress-inducing protocols to enrich protective protein expression are needed. However, synthetic biology offers a potential bypass: by engineering bacteria, yeast, or probiotics to produce tardigrade-derived molecules, researchers may avoid the need to culture the animals directly (Han et al., 2023; Sanchez-Martinez et al., 2024; Carvalho et al., 2025).

Delivering large biomolecules such as Dsup into human cells is another major barrier. Strategies under investigation include fusion proteins with nuclear localization signals, nanoparticle carriers, liposomes, and exosomes tagged with cell-penetrating peptides (Maier et al., 2012; Kirtane et al., 2025; Shim, 2025). Despite promising proof-of-concept in murine models, the clinical translation of Dsup is challenged by its immunogenicity, which may compromise efficacy upon repeated administration, its transient expression window, and reliance on localized delivery, necessitating further optimization for human use (Lim et al., 2023). Similar hurdles exist for other candidate therapies. DNA-targeted nitroxides suffer from an incompletely resolved mechanism and insufficient *in vivo* evidence (Huang et al., 2024), while the efficacy of metabolic agents like NAD^+^ precursors and pigments like betalains is highly dependent on precise dosing and long-term safety profiles. For structurally complex molecules, medicinal chemistry approaches, such as distilling key functional motifs or developing optimized analogs, will be crucial to improve drug-like properties (Kassab, 2025).

The unique nature of these non-human proteins, especially those that interact with DNA, also invites intense regulatory scrutiny. They cannot be adequately assessed within existing frameworks alone and will demand a distinct regulatory paradigm built upon pathways for biologics and gene therapies. A cornerstone of this paradigm will be a definitive risk-benefit profile, which is critically dependent on the application; use in patients or disaster scenarios may warrant greater risk, whereas the threshold for prophylactic use in healthy populations must be set exceedingly high.

These regulatory challenges are compounded by paramount biosafety concerns. Beyond the persistent issue of immunogenicity, the non-specific DNA-binding of proteins like Dsup poses an insidious risk of off-target interactions. This could occlude transcription factor binding sites or impede repair complexes, potentially leading to transcriptional dysregulation and genomic instability. It is therefore imperative that preclinical models verify no adverse interference with fundamental DNA metabolism and that long-term carcinogenicity studies are conducted to ensure radioprotection does not come at the cost of increased tumorigenesis later in life.

These scientific and safety considerations are inextricably linked to broader ethical dilemmas. The strategy of creating human-tardigrade chimeric constructs provokes deep-seated questions concerning biological identity and the ethical limits of cross-kingdom genetic integration. Moreover, deploying these interventions prophylactically in healthy populations conflates therapy with human enhancement, which could precipitate issues of social equity and coercion.

Despite these challenges, the outlook is highly encouraging. Interdisciplinary collaboration between radiobiologists, molecular biologists, chemists, engineers, and clinicians will be crucial for progress. Synthetic biology platforms may allow expression of Dsup or related genes in engineered probiotics for gut radioprotection. High-resolution structural studies of Dsup–nucleosome complexes, predictive pharmacokinetic models for mRNA/protein clearance, and scalable GMP-grade manufacturing pipelines are all on the near-horizon. Ethical frameworks for deploying these technologies—especially in healthy individuals, such as astronauts—must evolve in parallel with technical capabilities.

The potential applications of tardigrade-based radioprotectants are broad and transformative. In oncology, localized delivery of protective agents during radiotherapy could dramatically reduce treatment-related toxicity without compromising tumor control. Dsup mRNA gels or rectal formulations, for example, could shield mucosal linings during head, neck, or pelvic radiotherapy, thereby improving quality of life and enabling more aggressive treatment regimens. In space medicine, these agents could be administered prophylactically or in response to radiation events, protecting astronauts during long-duration missions from cumulative DNA damage. Options might include oral radioprotective supplements, implantable slow-release systems, or transient gene therapies to express protective proteins in critical tissues.

Tardigrade-inspired interventions may also serve in emergency medicine. Stockpiled formulations—ideally oral or injectable—could be rapidly deployed after nuclear accidents or radiological attacks to reduce acute radiation syndrome risk in exposed populations. Similarly, these protectants might be used to mitigate ongoing radiation damage post-exposure, aiding hematopoietic and gastrointestinal recovery. Beyond radiation, tardigrade-derived molecules hold promise in preserving genomic stability under oxidative or mechanical stress. Applications could span organ transplantation, chemotherapy-induced toxicity, biologic drug production, and even DNA preservation during laboratory imaging procedures.

Looking further ahead, one can envision tardigrade-based solutions being integrated into biosuits, spacecraft materials, or even as part of astronaut genome engineering in speculative scenarios. Although human germline modification remains ethically fraught and technologically premature, reversible somatic gene therapies or periodic infusions of Dsup-encoding nanoparticles might eventually become a reality for spacefarers. Studying tardigrade biology also advances our understanding of life’s boundaries, potentially guiding the search for life on Mars or Europa and inspiring materials science innovations.

Ultimately, the development of tardigrade-inspired radioprotectants illustrates the power of bioinspiration: learning from nature’s evolutionary achievements to solve human health challenges. While total immunity to radiation remains beyond reach, conferring partial resilience—borrowing molecular strategies from one of Earth’s most extreme survivors—could reshape how we approach cancer treatment, disaster preparedness, and human exploration of the cosmos.

## 5 Conclusion

Tardigrades—nature’s paradigm of extreme resilience—offer a compelling blueprint for reimagining radioprotection. Their multifaceted strategy of chromatin shielding, free-radical quenching, hyper-efficient DNA repair, and metabolic reprogramming yields a ≥10-fold survival advantage over current pharmacologic standards. By decoding and distilling these mechanisms into druggable formats—recombinant or cell-penetrating proteins, peptidomimetics, mRNA payloads, and biomimetic nanomaterials—biotechnology can marry evolutionary wisdom with modern engineering. Progress is already tangible: Dsup-expressing mammalian cells show markedly reduced double-strand breaks, while early animal studies demonstrate improved post-irradiation survival. Yet translational hurdles remain—scalable tardigrade cultivation, macromolecule stability, immunogenicity, and targeted delivery all demand interdisciplinary solutions spanning extremophile biology, chemical biology, materials science, and clinical oncology. With rapid advances in synthetic biology, computational protein design, and high-throughput screening, these obstacles are surmountable. The first tardigrade-inspired candidates could soon enter clinical or space-flight trials, heralding a new generation of radioprotectants that safeguard patients undergoing radiotherapy, astronauts venturing beyond Earth’s magnetosphere, and populations facing nuclear contingencies. In transforming the endurance secrets of a millimeter-sized “water bear” into life-saving therapeutics, humanity stands to bear the unbearable—turning an evolutionary curiosity into a cornerstone of innovative radiation medicine.

## Data Availability

The original contributions presented in the study are included in the article/supplementary material, further inquiries can be directed to the corresponding authors.

## Abbreviations

Dsup, Damage suppressor; TDR1,Tardigrade DNA-Repair protein 1; ROS, Reactive-oxygen-species; IDPs, Intrinsically disordered proteins; LEA, Late-embryogenesis abundant; DOPA, Dihydroxyphenylalanine; UV, Ultra-violet; CAHS, Cytosolic Abundant Heat Soluble Protein; MAHS, Mitochondrial Abundant Heat Soluble Protein; SAHS, Secreted Abundant Heat Soluble Protein; GMP, Good Manufacturing Practice; BCS1, Ubiquinol-cytochrome c reductase (bc1) synthesis; NDUFB8, NADH dehydrogenase (ubiquinone) 1 beta subcomplex subunit 8.
